# Ionic Mechanisms of Two-Pore Channel Regulation of Vesicle Trafficking

**DOI:** 10.3390/cells15020194

**Published:** 2026-01-20

**Authors:** Heng Zhang, Michael X. Zhu

**Affiliations:** 1 Institute of Molecular Physiology, Shenzhen Bay Laboratory, Shenzhen 518132, China; 2 Department of Integrative Biology and Pharmacology, McGovern Medical School, The University of Texas Health Science Center at Houston, Houston, TX 77030, USA

**Keywords:** TPCN1, TPCN2, calcium signaling, sodium efflux, fission, fusion, vesicle trafficking, tubulation, osmosis, acidic organelle

## Abstract

**Highlights:**

**What are the main findings?**

- Two-pore channels (TPC1 and TPC2) are intracellular channels activated by NAADP and PI(3,5)P_2_ to release Ca^2+^ and Na^+^ from endosomes and lysosomes.
- TPC-mediated effluxes of Ca^2+^ and Na^+^ play distinct roles in intracellular vesicle trafficking.

**What are the implications of the main findings?**

- The dual regulation by NAADP and PI(3,5)P_2_ explains the diverse functions of TPCs in physiology and disease.
- Both Ca^2+^- and Na^+^-mediated effects need to be considered when evaluating the mechanism of TPC-regulation of cellular function.

**Abstract:**

The endolysosomal system plays a pivotal role in cellular function. Before reaching lysosomes for degradation, the endocytosed cargoes are sorted at various stages of endosomal trafficking for recycling and/or rerouting. The proper execution of these processes depends on tightly regulated ion fluxes across endolysosomal membranes. Recent studies have demonstrated the importance of two-pore channels (TPCs), including TPC1 and TPC2, in endolysosomal trafficking. These channels are expressed in the membranes of distinct populations of endosomes and lysosomes, where they respond to nicotinic acid adenine dinucleotide phosphate (NAADP) and phosphatidylinositol 3,5-bisphosphate [PI(3,5)P_2_] to conduct Ca^2+^ and Na^+^ release from these acidic organelles. Here, we discuss the potential implications of Ca^2+^ and Na^+^ fluxes mediated by TPCs across endolysosomal membranes in the physiological and pathophysiological functions of these organellar channels.

## 1. Introduction

The biomembrane serves important roles in separating not only the extracellular and intracellular environments but also the different cellular compartments, namely the subcellular organelles, within the cytoplasm. This allows water-soluble substrates, such as ions, nutrients, and metabolites, to be segregated for efficient processing in response to various functional demands, in addition to preventing random mixing. For water-soluble nutrients and metabolites, there are three major mechanisms for them to accumulate or be extruded from a certain cellular compartment: (i) enzymatic synthesis and/or degradation, (ii) vesicular transport, and (iii) protein-aided transport across the membrane. All these require proper ion concentrations on the respective sides of the membrane. First, since protein folding is highly influenced by the ionic strength of the environment, proper ionic concentrations are essential for all enzymatic activities, not to mention that many enzymes are dependent on specific concentrations of H^+^ (pH), Ca^2+^, Cl^−^, or other ions, for their function. As will be elaborated later in this review, vesicular transport is also highly dependent on Ca^2+^, Na^+^, and Cl^−^. Moreover, protein-aided transport can occur through either facilitated diffusion or secondary active transport, both of which are mediated by carrier proteins that traverse the membrane. Specifically, secondary active transport depends on either Na^+^ or H^+^, which induces a conformational change in the membrane protein by directly binding to it, thereby flipping the substrate from one side of the membrane to the other. In this case, energy stored in ion gradients is transduced into secondary active transport, and the concentration gradient of Na^+^ or H^+^ strongly influences the rate, and sometimes even direction, of substrate transport. Thus, ionic gradients and concentrations across the biomembrane, as well as their changes, strongly impact the function and homeostasis of the cell [1].

Ions are an indispensable part of life, as they play crucial roles in every aspect of cellular function. Asymmetric concentrations of biological ions, mainly Na^+^, K^+^, Cl^−^, Ca^2+^, and H^+^, across biomembranes are established through the actions of ion pumps and ion exchangers, creating ionic gradients that are utilized by ion channels and solute transporters to regulate cell function. As a classic example, the membrane potential is determined by the asymmetric ion concentrations across the membrane and the selective permeation of ions through ion channels located within the membrane. At the plasma membrane (PM), the large K^+^ gradient—high inside and low outside because of the action of Na^+^/K^+^-ATPases, or Na^+^ pumps—and the relatively high K^+^ permeability—due to opening of leak K^+^ channels—are responsible for the negative potentials under resting conditions [2]. In the mitochondrial inner membrane, a strong negative potential (~−150 mV) is set by the large proton gradient created by the electron transport chain that pumps protons from the mitochondrial matrix to the intermembrane space [3]. By analogy, other membranous organelles should also have resting membrane potentials, although the values and mechanisms underpinning them remain largely mysterious.

Although either a change in the ionic gradient across or a shift in ion permeability of the membrane can alter the membrane potential, it is typically the latter that serves a cell signaling function, especially at the PM. The best examples are found in excitable cells such as neurons, muscles, and endocrine cells, where an increase in Na^+^ permeability causes membrane depolarization that serves as a trigger for action potential firing, muscle contraction, and hormone secretion, respectively. These complex processes involve several types of ion channels, from ligand-gated non-selective cation channels to voltage-gated Na^+^ and Ca^2+^ channels, each contributing to a unique aspect of the signaling process [4]. Particularly, the Ca^2+^ channels mediate Ca^2+^ influx from the extracellular space to the intracellular milieu, leading to a rise in cytosolic Ca^2+^ concentration ([Ca^2+^]_c_), also known as Ca^2+^ signal because Ca^2+^ serves as a second messenger to regulate the function of many effector proteins in a spatiotemporal manner. It should be emphasized that Ca^2+^ signal is the ultimate trigger that induces contraction and secretion in muscles and endocrine cells. This principle also applies to neurons, as action potentials eventually trigger Ca^2+^ influx at the axon terminals through voltage-gated Ca^2+^ channels to cause the release of neurotransmitters. These depolarization-driven processes, which employ asymmetrical ion distributions across the membrane, sequential changes in ion permeation through multiple ion channel types, and Ca^2+^ signaling to drive contraction and secretion, are termed excitation-contraction and excitation-secretion coupling, respectively, representing hallmarks of modern physiology [5,6]. On the other hand, various K^+^ channels and Cl^−^ channels participate by countering the depolarizing effect of Na^+^ and Ca^2+^ channels, providing the brake to allow precise control of the process.

In a different context, ion channels are involved in cell volume regulation in processes referred to as regulatory volume decrease (RVD) and regulatory volume increase (RVI) [7]. RVD occurs when cells are exposed to a hypotonic environment. Cells first swell due to water uptake driven by osmosis. This activates volume-regulated anion channels and Ca^2+^-activated K^+^ channels, reducing the concentrations of ions and osmolytes in the cytosol. Consequently, water also flows out of the cell, allowing it to return to its original volume. In RVI, cells first shrink when exposed to a hypertonic environment due to water loss, again by osmosis. Then, a number of ion transporters and channels, mainly Na^+^ channels, are activated to bring extra ions and osmolytes into the cell. This increases the intracellular osmolarity, allowing water uptake to restore the cell volume. Additionally, ion channels are also important for water secretion and absorption in the epithelium, employing a similar ion flux-leads-and-water-follows mechanism as in RVD and RVI, except for the presence of a paracellular pathway driven largely by the transepithelial potential set up by transcellular ion transport [8,9]. Thus, the efflux of Cl^−^ through the cystic fibrosis transmembrane conductance regulator (CFTR) and Ca^2+^-activated Cl^−^ channels at the apical membrane drives paracellular Na^+^ leak and subsequent water secretion through both transcellular and paracellular pathways. Conversely, the epithelial Na^+^ channel (ENaC) conducts apical Na^+^ influx, which in turn promotes paracellular Cl^−^ transport and then water absorption. Therefore, by rapidly changing the concentrations of major ions, like Na^+^, K^+^, and Cl^−^, across the membrane, ion channels facilitate water transport through osmosis. This feature is different from the channels involved in membrane potential regulation, which are thought not to have a significant impact on the concentrations of these major ions.

Collectively, the above examples highlight the primary contributions of ion channels to cell function, membrane potential regulation, Ca^2+^ signaling, and water transport. Historically, our understanding of ion channels and their physiological significance has been gained from studying PM-localized channels, especially those expressed in excitable cells. More recent studies, however, have uncovered the importance of ion channels not only in non-excitable cells, but also within intracellular membranes such as those circumscribing the endoplasmic reticulum (ER), mitochondria, and endosomes and lysosomes. Although clear differences exist between the classical, well-studied channels in excitable cells and the newer channels found in non-excitable cells and intracellular membranes in terms of activation and regulation mechanisms, kinetics, and (patho)physiological roles, the general principle still applies that these channels primarily exert their actions by affecting membrane potential, Ca^2+^ signaling, and water transport. In this review, we focus on the discovery and function of two-pore channels (TPCs), which are found mainly associated with endosomes and lysosomes, collectively referred to as endolysosomes.

## 2. TPCs Are Members of the Voltage-Gated Cation Channel Superfamily

**Figure 1:**
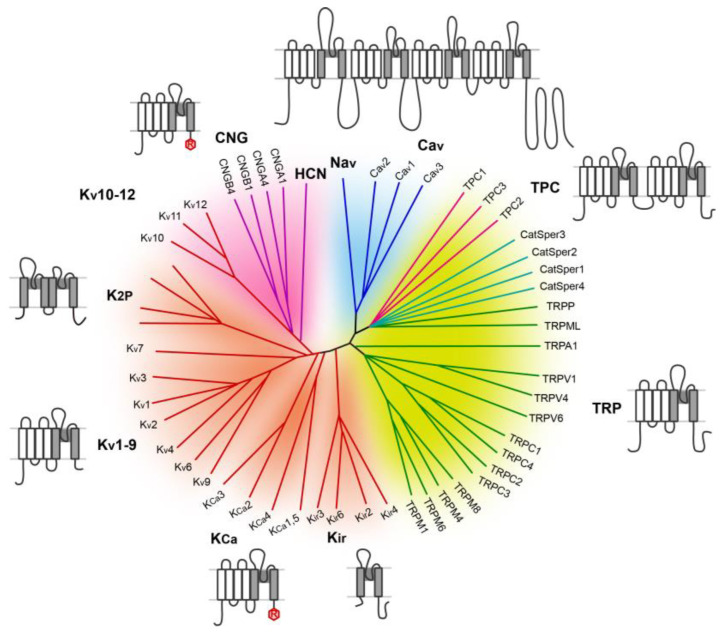
The voltage-gated cation channel superfamily. The phylogenetic tree was modified according to ref. [10], highlighting the relation among Nav, Cav, two-pore channels (TPCs), CatSper (Cation channels of Sperm), and TRP (Transient Receptor Potential) channels. Four-domain channels (Cav and Nav) are shown as blue branches, TPCs are shown in magenta branches, Catspers are shown in teal branches, TRP channels are shown as green branches, potassium-selective channels are shown as red branches, and cyclic nucleotide-gated channels (CNG and HCN) are shown as purple branches. Background colors separate the channels into related groups: light blue, Cav and Nav; light green, TPC, CatSper, and TRP channels; light red, potassium channels, with the exception of Kv 10–12, which contain a cyclic nucleotide-binding domain and are more closely related to CNG and HCN channels; purple, Kv 10–12 channels, CNG and HCN channels. Diagrams show the single-subunit membrane topology for the major subfamilies, open area: S1–S4 voltage-sensing domain (VSD) and voltage-sensor-like domain (VSLD), shaded area: S5–P–S6 or TM1–P–TM2 pore-domain.

Voltage-gated cation channels, including voltage-gated K^+^ (Kv) channels, voltage-gated Na^+^ (Nav) channels, voltage-gated Ca^2+^ (Cav) channels, and many non-selective cation channels, constitute the largest family of ion channels, with over 100 members (Figure 1). Although not all members of this large family are voltage-gated, they share sequence homology and a similar structural organization. They also represent the earliest ion channels known to physiologists and the best characterized channel types [10].

The basic functional unit of this channel family consists of eight transmembrane (TM) helices formed by a four-subunit (tetrameric) complex, with each subunit contributing two TM helices, as represented by the inwardly rectifying K^+^ channels (Kir’s) (Figure 1). A Kir subunit also contains an N-terminus before the first TM helix (TM1) and a C-terminus after the second TM helix (TM2), both located at the cytoplasmic side. In the tetramer, TM2’s face each other in a 4-fold symmetry, creating a water-filled tunnel for the passage of ions, which is referred to as the pore. The four TM2 helices intercross at their C-terminal sides to form the so-called four-helix bundle, which represents the narrowest part of the pore, also referred to as the inner gate due to its location in the inner leaflet of the PM lipid bilayer. When channels are closed, the inner gate has a diameter smaller than that of the permeant ions, preventing their passage. The opening of the channel typically involves a rotation of the TM2 helix, which expands the inner gate diameter large enough for the ions to pass. The selection of the ions is made at the selectivity filter (SF) located at the level of the outer leaflet of the bilayer. The SF is a part of the pore loop, or P-loop, situated between the two TM helices, which enters the membrane only halfway without crossing it completely. In K^+^ channels, the SF exhibits a conserved T-V-G-Y-G signature sequence, which faces each other in the tetrameric complex to form a passage that only accommodates dehydrated potassium ions. In other channels, this sequence is altered to allow selection for Na^+^, Ca^2+^, or all cations, without or with partial dehydration. The SF region can be gated, meaning closed at rest and open in response to stimuli; in such a case, it is also referred to as the outer gate [11].

In two-pore K^+^ (K2P) channels, two homologous 2-helix units are contained in a single polypeptide (Figure 1), indicative of a duplication of a Kir-like gene in early evolution, which later expanded to as many as 15 K2P-coding genes in the human genome. As a result, a K2P channel is made of two subunits, each containing two pore-forming domains. These dimeric channels have a two-fold symmetry. They also possess a unique extracellular helical cap not seen in other channels. Other than these, the TM architecture of K2P channels is similar to that of Kir channels [12].

The majority of the voltage-gated channel family members, however, have another domain composed of an additional four TM helices that preceded the two pore-forming TM helices (Figure 1). These are often referred to as S1–S4, where S stands for segment, and the two pore-forming TM helices are called S5 and S6. While the S5 and S6 segments, as well as the P-loop in between, share sequence homology with TM1, TM2, and the P-loop, respectively, of the Kir channels, the S1–S4 domain is evolutionarily related to the Hv1 voltage-gated proton channel and the voltage sensor domain of voltage-sensitive phosphatases (VSPs), suggesting the occurrence of a fusion event between a voltage sensor and a pore-forming protein in early evolution. Supporting this idea, the S1–S4 TM helices form the voltage-sensing domains (VSD) of the classical voltage-gated channels, including Kv, Nav, and Cav channels. Particularly, the S4 segment of the VSD contains an array of positively charged amino acids repeated every three residues, which represents the hallmark of the VSD [13]. However, it remains unclear to what extent the VSD contributes to the voltage sensing of transient receptor potential (TRP) channels, which are often weakly voltage-sensitive or have no voltage sensitivity at all, despite all containing the equivalent S1–S4 TM helices, typically described as voltage-sensor-like domains (VSLDs) [14].

The 6-TM unit, S1–S4 from VSD or VSLD and S5–P–S6 from the pore domain, constitutes the basic building block of all Kv, Nav, Cav, cyclic nucleotide-gated channels, CatSper, and TRP channels. The channel is composed of four such units, or subunits, with the S5–P–S6 domains situated in the center assembled in a similar fashion to the Kir channels, and the S1–S4 domain placed in the periphery. In most of these channels, they are “domain-swapped”, in that the S1–S4 domain of one subunit interfaces with the S5–P–S6 domain of a neighboring subunit. In Nav and Cav channels, however, the pore-forming subunit contains four such 6-TM units in a single polypeptide in tandem, indicating that an ancient 6-TM domain-encoding gene probably underwent two rounds of duplication during early evolution. Interestingly, each of the four 6-TM domains, or at least three of them, in Nav and Cav channels still contains a functional VSD, characterized by the array of positively charged residues in the respective S4 segment. Unlike Kv channels, the key amino acids at the center of the SF are asymmetric in Nav and Cav channels due to sequence divergence among the four 6-TM repeats, which is thought to be crucial for their selective permeation of partially dehydrated Na^+^ and Ca^2+^ ions, respectively.

If two rounds of duplication occurred for the creation of voltage-gated Na^+^ and Ca^2+^ channels, then an intermediate product with two 6-TM units contained in one polypeptide should have existed during evolution. Indeed, this is where two-pore channels (TPCs, also known as two-pore domain channels or two-pore segment channels) fit within this large superfamily (Figure 1). The name “TPC” was given by researchers who first reported the sequence of the rat TPC1 gene after two-pore K^+^ channels, to emphasize that it likely encodes two pore-forming domains, each predicted to have 6-TM helices that share some limited homology with Nav and Cav [15]. The fact that TPC genes are also found in green algae and vascular plants supports the notion that these ancient genes may date back to the time of unicellular organisms before the divergence between animals and plants. Indeed, the demonstration that *Arabidopsis* TPC1 mediated Ca^2+^ release from the plant’s vacuole represents the first functional characterization of the TPCs [16]. The function of animal TPCs was not unveiled until several years later, when three research groups independently reported that mammalian TPC1 and TPC2 conducted Ca^2+^ release from endolysosomes in response to nicotinic acid adenine dinucleotide phosphate (NAADP) [17,18,19], a potent Ca^2+^ mobilizing messenger known to act at lysosome-related acidic organelles [20]. This marked the beginning of the era of structural and functional characterization, as well as pathophysiological studies, of the TPCs.

## 3. Basic Structural Features and Subcellular Distributions of TPCs

**Figure 2:**
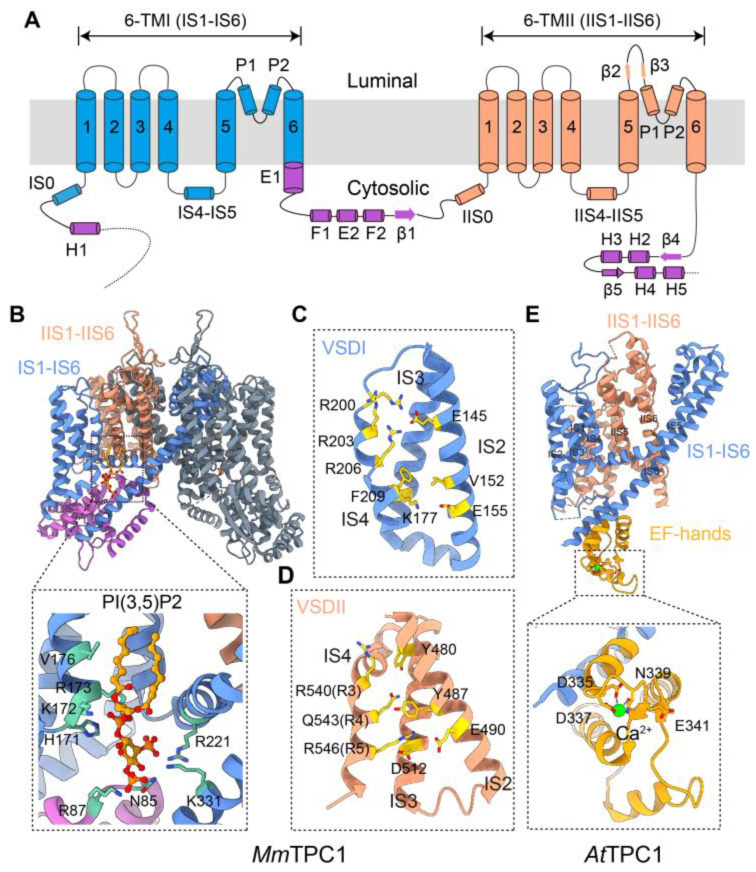
Structural features of mammalian TPC1 (*Mus musculus*, *Mm*TPC1) and plant TPC1 (*Arabidopsis thaliana*, *At*TPC1). (**A**) Topology and domain arrangement of an *Mm*TPC1 subunit. (**B**) Side view of *Mm*TPC1 channel dimer (PDB ID: 6C9A). 6-TM domain I (IS1–IS6), 6-TM domain II (IIS1–IIS6), and other domains from one subunit are shown in blue, salmon, and purple, respectively. The other subunit is shown in slate gray. Zoomed-in view shows the PI(3,5)P_2_-binding pocket. (**C**,**D**), Zoomed-in views of voltage-sensing domain I (VSDI) and VSDII of *Mm*TPC1, with key residues labeled in gold. (**E**) Side view of an *At*TPC1 channel subunit (PDB ID: 5E1J). 6-TM domain I (IS1–IS2), 6-TM domain II (IIS1–IIS6), and EF hands are shown in blue, salmon, and orange, respectively. Zoomed-in view shows the Ca^2+^-binding pocket in *At*TPC1.

Each subunit of the TPC contains two 6-TM units connected by an interdomain loop that faces the cytoplasm. Both the N- and C-termini are also exposed to the cytoplasmic side (Figure 2A). More recent cryo-EM structures of *Arabidopsis* TPC1, mouse TPC1, human TPC2, and zebrafish TPC3 confirm the early prediction that the channel is composed of a dimer made up of two TPC subunits, with an overall architecture similar to that of the Kv, Nav, and Cav channels [21,22,23,24,25]. Thus, a TPC dimer exhibits pseudo four-fold symmetry, with each subunit contributing two pore domains, IS5–IP–IS6 and IIS5–IIP–IIS6, to form the ion-conducting pore in the center, and two VSDs, IS1–IS4 and IIS1–IIS4, that embrace the pore in the periphery, again in a domain-swapped manner (Figure 2B). At high resolutions, the symmetry is two-fold due to the sequence divergence between IS5–IP–IS6 and IIS5–IIP–IIS6, as well as that between IS1–IS4 and IIS1–IIS4; however, this may be crucial for the ion selectivity and functional regulation of the channel. Although heteromerization between mammalian TPC1 and TPC2 has been postulated [26], TPC2 did not co-purify with TPC1 from mouse kidney, which endogenously expresses high levels of these proteins [27]. In addition, high-resolution structural studies thus far have only reported homomeric TPCs. Also, the relatively extensive distributions of TPC1 in the endosomes and TPC2 in the lysosomes [17,27,28] make it unlikely that the two channels coexist in the same organelle, although temporary colocalization upon vesicle fusion, thereby heteromerization through subunit disassembly and reassembly, cannot be completely ruled out.

Three TPC paralogs (*TPCN’s* for gene names) are found in animals. Their origin can be traced back to a time before the separation of fungi and animals, as all three *TPCN* genes are found in unicellular organisms such as choanoflagellates and other distant protists [17,29]. In addition, these protists also have an additional branch of TPC-related (or *TPCR*) genes, which are not found in animals. However, TPCs do not appear to be essential for survival in animals, as their genes are absent from the model species *Caenorhabditis elegans* and *Drosophila melanogaster*. In fact, they are completely missing in the *Diptera* order (flies and mosquitoes). In other insects, e.g., silkworms, bees, and beetles, only TPC1 exists [30]. In *Deuterostomia*, including echinoderms (sea urchins) and all vertebrates, all three *TPCN* genes are typically preserved. However, certain species of rodents (mice and rats) and primates (monkeys, apes, and humans) have lost the *TPCN3* gene. The reason for the low conservation of the *TPCN* genes in animal species is unknown. Most plants, especially higher plants, have just one TPC, also named TPC1. However, the plant TPC1 is equally distant from the animal TPC1, TPC2, and TPC3, suggesting that the divergence of the three *TPCN*s occurred later after the separation of plants, fungi, and animals. Notably, the three animal TPCs are quite distant from each other, consistent with the notion that their divergence occurred early in the evolution. At least in the commonly studied species, no further duplication was found for the individual TPC paralogs. This is somewhat surprising, as more recent duplications leading to two or more subtypes within a subgroup of voltage-gated cation channels, such as Kv1.x channels, Cav1.x channels, Nav1.x, and TRPM channels, are common in this superfamily. This lack of diversification could suggest that each of the TPC subtypes is dedicated to a general basic cellular function, relatively independent of the cell types in which it is expressed. This view is consistent with the selective expression of TPC1 in early and recycling endosomes and TPC2 in lysosomes, where they are involved in intracellular vesicle trafficking. Currently, less is known about the precise subcellular localization of TPC3; however, there is evidence that it tends to be expressed in recycling endosomes, lysosomes, and the PM [28].

With two 6-TM domains arranged in tandem and two subunits combined to form a channel that displays similar architecture to Nav and Cav channels, as well as sequence homology, although relatively low, to Nav and Cav channels, it would be reasonable to assume that Nav and/or Cav might have evolved by linking two *TPCN* genes together. However, it is questionable whether this was the case. First, TPCs are primarily localized in subcellular organelles, unlike the PM localization of the Nav and Cav channels. Second, and perhaps more importantly, only the second 6-TM domain of the TPC is voltage-gated; the first 6-TM domain of the mammalian TPC is gated by a lipid, mainly phosphatidylinositol 3,5-bisphosphate [PI(3,5)P_2_] (Figure 2B–D) [23,24]. In electrophysiological recordings, mammalian TPC1 and TPC3 show clear co-dependence on PI(3,5)P_2_ and voltage [31,32]. The voltage dependence of TPC2, however, is not apparent because the IIS4 voltage sensor is permanently locked in the “up” position, meaning that domain II is “constitutively activated” and the channel is primed to open as soon as PI(3,5)P_2_ binds to domain I [24]. Thus, instead of arising from the duplication of two voltage-gated 6-TM units, TPCs probably derived from a combination of one lipid-gated 6-TM unit and one voltage-gated 6-TM unit. This evolutionary route could be traced to TRP channels, which are often gated or at least modulated by phosphatidylinositol 4,5-bisphosphate [PI(4,5)P_2_], through direct binding at the VSLD region [33]. Plant TPC1, while sharing a common mechanism of voltage-gating through the 6-TM domain II, is not known to be activated by PI(3,5)P_2_. Instead, the co-activator is Ca^2+^, which binds to the EF hands located in the intracellular loop between the two 6-TM domains (Figure 2E) [21]. Based on a sequence alignment at selectivity filters, protist TPCRs may be closer to Cav channels than TPCs [29] and might represent the ancestral two-pore domain structure that gave rise to Cav and Nav channels through duplication. However, it is unclear whether both domains of the TPCRs contain bona fide VSDs.

## 4. Function and Regulation of Mammalian TPCs

Endolysosomes are classically recognized as acidic organelles owing to their low luminal pH, maintained by the vacuolar H^+^-ATPase (V-ATPase). Given the high enrichment of TPCs in these organelles and the seminal demonstration that plant TPC1 mediates Ca^2+^ efflux from vacuoles—organelles evolutionarily related to animal lysosomes—it was natural to hypothesize that mammalian TPCs act as Ca^2+^ release channels on endosomes and lysosomes. Moreover, it has long been known that NAADP, a very potent Ca^2+^ mobilizing second messenger, targets lysosome-related acidic organelles [20]. Therefore, early investigations focused on testing whether TPC1 and TPC2 might serve as NAADP-responsive Ca^2+^ channels. Indeed, overexpression of either isoform markedly potentiated NAADP-evoked cytosolic Ca^2+^ elevations, whereas genetic deletion or knockdown of these isoforms abolished such responses [17,18,19]. The endolysosomal origin of the Ca^2+^ signal was established by eliminating NAADP-evoked responses after dissipating the acidic stores with the V-ATPase inhibitor bafilomycin A1 or disrupting the integrity of lysosomal membrane with glycyl-L-phenylalanine 2-naphthylamide (GPN), a lysosomotropic agent [17]. This contrasts with ER-derived signals, which are instead abolished by inhibiting SERCA (SarcoEndoplasmic Reticulum Ca^2+^-ATPases) with thapsigargin.

NAADP has long been shown to arise from NADP through a base exchange reaction catalyzed by the NAD-glycohydrolase/ADP-ribosyl cyclase, CD38 [34,35], and, more recently, SARM1 [36]. In addition, NADPH oxidases NOX and DUOX can also convert NAADPH to NAADP [37]. Physiologically, NAADP production and its stimulation of Ca^2+^ mobilization from acidic stores are an integral part of receptor signaling elicited by cholecystokinin, interleukin-8, endothelin, and several other hormones and chemokines, as well as processes like glucose-induced insulin secretion from pancreatic β cells, fertilization, and sensing of nutrients, e.g., nicotinic acid [38]. Therefore, the identification of TPC1 and TPC2 as the long-sought-after NAADP receptors is of great significance to understanding these physiological processes. Although the primary effect of NAADP signaling is Ca^2+^ mobilization from endolysosomes, which produces local Ca^2+^ transients, under certain conditions, these local Ca^2+^ events can trigger secondary Ca^2+^ release from the ER through a mechanism known as Ca^2+^-induced Ca^2+^ release (CICR), because both inositol 1,4,5-trisphosphate (IP_3_) receptors and ryanodine receptors are exquisitely sensitive to cytosolic Ca^2+^. This not only amplifies the Ca^2+^ signal but also transforms it from discrete local events at the lysosomal nanodomains into global Ca^2+^ waves in the whole cell [17,30]. Evidently, this transformation is facilitated much more robustly by the overexpression of TPC2 than by TPC1 [17,28].

Recently, Yuan and colleagues provided high-resolution evidence of this local-to-global transition, showing that TPC2-mediated Ca^2+^ sparks transform into a global increase in [Ca^2+^]_c_ upon engagement of IP_3_ receptors [39]. This establishes a mechanistic framework in which lysosomal Ca^2+^ release via TPC2 serves as the initiating event that entrains ER Ca^2+^ stores to generate global signals. However, whether the TPC-generated Ca^2+^ signals can globalize depends on the amplitude and frequency of these local signals, their proximity to the ER, the level of IP_3_, and the availability and density of IP_3_ and ryanodine receptors. On the other hand, in the common pathway involving receptor activation of phospholipase C (PLC), the TPC2-generated Ca^2+^ events are particularly effective in boosting the responses elicited by low agonist concentrations [39]. Thus, the interplay between the endolysosomal and ER Ca^2+^ release channels and their corresponding stores plays a crucial role in shaping the Ca^2+^ signatures and their downstream signaling pathways.

The Ca^2+^ signals are thought to underlie many of the TPC-dependent cellular processes—including endocytosis, vesicular trafficking, autophagosome–lysosome fusion, and lysosomal exocytosis—each requiring spatially and temporally controlled Ca^2+^ flux, arising presumably from individual organelles. The local Ca^2+^ nanodomain near the mouth of the TPC2 pore has been estimated to reach a concentration above 40 µM, which quickly dissipates to ~1–2 µM in a short distance [40]. Thus, when not globalized, the TPC-generated Ca^2+^ signals serve pivotal and regionally restricted roles, underscoring their multifaceted effects in different cellular and physiological contexts. The high micromolar Ca^2+^ concentrations at these nanodomains enable TPC to directly regulate proteins with a relatively low Ca^2+^ affinity, although the identity of these proteins remains largely unexplored. In addition to targeting IP_3_ receptors and ryanodine receptors, calcium ions generated through TPC2 likely also exert their effects through calmodulin (CaM). This might underlie the effect of TPC2 activation on nuclear translocation of TFEB (transcription factor EB) [41], analogous to the CaM-calcineurin-mediated action downstream from another lysosomal channel TRPML1 [42]. However, direct evidence for TPC2 regulation of lysosome biogenesis via this pathway is still lacking. It has been suggested that the local Ca^2+^ nanodomains produced by TPC2 and TRPML1 do not overlap [40], and these channels are involved in distinct physiological processes [43]. In endocytosis, Ca^2+^ arising from TPCs has been linked to dynamin activation [44,45], and this regulation was found to occur through dephosphorylation of dynamin by CaM-calcineurin, instead of a direct binding of dynamin by Ca^2+^ [44]. PKC-βII may be another Ca^2+^-binding protein activated in a TPC2-dependent fashion; however, this regulation also appears to be indirect and involves TPC2 regulation of Orai1 expression and Ca^2+^ influx through Orai1, a Ca^2+^-selective channel expressed on the PM [46]. Another Ca^2+^-binding protein, synaptotagmin 7, has been shown to cooperate with TPC1 in antigen presentation of intracellular mycobacterial infections by MHC-related protein 1 [47]. Again, more direct evidence is needed for TPC1 regulation of synaptotagmin 7 through its Ca^2+^ release function. It should also be noted that because luminal Ca^2+^ potently influences lysosomal acidification, some TPC-dependent phenotypes may arise indirectly through pH-dependent mechanisms rather than through cytosolic Ca^2+^ elevations per se. For example, high TPC2 activity causes hyperacidification of melanosomes, which impairs melanin synthesis, leading to a lighter skin color [48,49].

As ion channels, TPCs must be characterized electrophysiologically, but their intracellular localization made such studies difficult in the early days. Several approaches were employed to overcome this challenge. First, purified TPC proteins reconstituted into planar lipid bilayers revealed NAADP-sensitive conductances permeant to K^+^, Ca^2+^, and H^+^ [50,51]. Second, engineered PM-targeted TPC2 channels enabled direct patch-clamp analysis, including inside-out recordings that permit cytosolic application of NAADP [52]. Third, overexpressed TPC1 and TPC3 display sufficient PM trafficking to allow conventional electrophysiological examinations [31]. A decisive advance came with the whole-endolysosome recording technique first developed by Haoxing Xu’s group for the study of TRPML1 channel [53,54]. This technique utilizes enlarged vacuoles found in cells treated with either vacuolin-1, a lysosome exocytosis inhibitor, or other agents that cause endolysosome enlargement through various mechanisms [55,56]. By manually breaking the PM with a sharp glass electrode, a vacuole can be released from the cell, allowing for direct access by a recording electrode. This way, both ionic currents and membrane potential across the endolysosomal membrane can be examined using the standard voltage- and current-clamp methods, respectively. However, when the whole-endolysosomal recording technique was initially applied to TPCs, no NAADP-evoked currents were detected. Instead, the channels showed a robust activation in response to PI(3,5)P_2_ [57,58], a phosphoinositide known to be enriched in the membranes of endosomes and lysosomes and previously shown to activate TRPML1 [54,59]. In addition, the channels show a pronounced preference for Na^+^ over K^+^, with only limited Ca^2+^ permeability [57,58]. Although later refinements of this technique enabled the detection of NAADP-evoked currents, these were markedly smaller than the currents evoked by PI(3,5)P_2_, often conditional on luminal ionic composition and holding potential, and not consistently reproduced across laboratories [60,61,62,63,64]. These observations intensified a long-standing debate regarding whether NAADP directly gates TPCs. To date, no evidence supports direct NAADP binding to TPC1 or TPC2; rather, NAADP appears to act through auxiliary proteins such as Lsm12 or JPT2 (HN1L), which may serve as adaptors that couple NAADP binding to conformational changes in TPCs [65,66,67].

**Figure 3:**
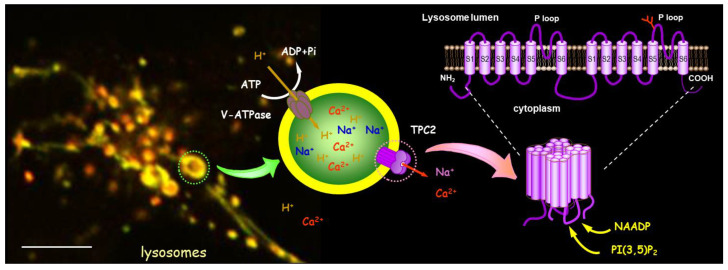
TPCs are endolysosomal cation channel coregulated by NAADP and PI(3,5)P_2_. Merged immunofluorescence image shows colocalization of HA-tagged human TPC2 (*green*) and LAMP2 (*red*) in a HEK293 cell. Diagram in the middle illustrates the dependence of the lysosome on V-ATPase for acidification and the function of TPC2 in Na^+^ and Ca^2+^ release. Diagram on the right displays the transmembrane organization of TPC and the assembled dimeric channel activatable by NAADP and PI(3,5)P_2_.

PI(3,5)P_2_ is produced from phosphatidylinositol 3-phosphate (PI3P) by the phosphoinositide 5-kinase PIKfyve (also known as Fab1 in yeast). This reaction is tightly regulated and known to respond to various stimuli, including osmotic stress, hormones, growth factors, changes in nutrient availability, etc. [68]. Thus, the above findings indicate that TPCs are endolysosomal cation channels coregulated by two endogenous agonists, NAADP and PI(3,5)P_2_, with broad physiological implications (Figure 3). Interestingly, TPC1 and TPC2 are inhibited by mTOR [58] and TMEM63 (also known as OCaR1) [69]. While the former is a metabolic regulator, the latter is a group of mechanosensitive cation channels, some of which also function as lipid scramblase [70]. Both protein groups target endolysosomes to exert their function, and their effects on TPCs further highlight the importance of these channels in cell physiology.

It is possible that NAADP (via NAADP-binding proteins like Lsm12 and/or JPT2) and PI(3,5)P_2_ stabilize distinct channel conformations, with NAADP favoring a more Ca^2+^-selective state and PI(3,5)P_2_ promoting a predominantly Na^+^-permeable mode. This idea gained support from the identification of two small-molecule TPC2 agonists, TPC2-A1-N and TPC2-A1-P, by the Grimm group. Based on Ca^2+^ imaging, whole-endolysosome recordings, and permeability estimates derived from reversal potentials, TPC2-A1-N was proposed to mimic NAADP by promoting a more Ca^2+^-selective open conformation, whereas TPC2-A1-P appeared to phenocopy PI(3,5)P_2_ in stabilizing a strongly Na^+^-selective state [71]. These findings suggest that TPC2 may exhibit at least two functionally distinct activation modes: a Ca^2+^-dominant mode optimized for Ca^2+^ nanodomain formation and localized Ca^2+^ regulated processes (not excluding the globalization via CICR and the functional consequences of such action, as described above), and a Na^+^-dominant mode with potential consequences on endolysosomal membrane potential, osmotic balance, and Na^+^-dependent transport processes.

The availability of TPC2-A1-N and TPC2-A1-P further enabled exploration of potential synergy between NAADP- and PI(3,5)P_2_-dependent gating mechanisms. Indeed, the two agonists produced supra-additive Ca^2+^ signals and ionic currents [72], and whole-endolysosome experiments revealed that a normally ineffective concentration of NAADP (1 nM) markedly potentiated PI(3,5)P_2_-evoked currents at equally low concentrations [64]. Whether this synergism is physiologically relevant remains unresolved. In mouse pulmonary artery smooth muscle cells, for example, TPC2 deletion abolished NAADP-evoked Ca^2+^ transients but left PI(3,5)P_2_ responses intact [63], suggesting parallel rather than obligatorily cooperative pathways. In other cell types, crosstalk between TPC2 and IP_3_ receptors preferentially involves signaling initiated by TPC2-A1-N over TPC2-A1-P, raising additional questions about the equivalence of these agonists with endogenous ligands. Furthermore, whether TPC2-A1-N and TPC2-A1-P truly recapitulate the functions of NAADP and PI(3,5)P_2_, respectively, remains unclear given their unrelated chemical structures. Unlike NAADP, TPC2-A1-N acts directly on TPC2, without the involvement of Lsm12 or JPT2. Similarly, the binding site and mechanism of action of TPC2-A1-P relative to PI(3,5)P_2_ remain unverified. Thus, although these synthetic agonists have greatly expanded the toolkit for studying TPC2, the extent to which they faithfully reproduce endogenous NAADP- and PI(3,5)P_2_-mediated activation requires further mechanistic clarification. It is also noted that a recent report claims that TPC2-A1-N can evoke Ca^2+^ signals independently of TPC2 [73].

## 5. Basic Mechanisms Underlying the Physiological and Pathophysiological Functions of Mammalian TPCs

Many physiological and pathophysiological functions have been linked to mammalian TPCs based on studies using *TPCN1* and *TPCN2* gene knockout and knockdown cells and animals, as well as pharmacological manipulations of these channels. These include pigmentation, neurodegeneration, cardiac function and dysfunction, metabolic disorders such as obesity and diabetes, immune function, cancer, and infection by enveloped viruses such as Ebola virus and SARS-CoV-2. Several recent review articles have provided detailed coverage of these functions [74,75,76,77]; therefore, they will not be repeated here. In essence, TPCs likely contribute to these functions due to their regulation of intracellular vesicle trafficking, which encompasses multiple steps of endocytosis, exocytosis, and autophagy. For example, TPCs are involved in virus infection and macrophage function by modulating phagocytosis and macropinocytosis, two specialized forms of endocytosis [40,62,78,79]. TPC1 regulates antigen presentation of infected tuberculosis bacteria by exerting an effect on the endosomal pathway of MHC-related protein 1 [47]. In melanoma, TPC2 has a negative effect on MHC-I-mediated tumor antigen presentation by promoting autophagic flux, and thereby degradation of MHC proteins [80]. This mechanism may be applicable to hepatocellular carcinoma as well, where inhibiting TPC2 stabilizes MHC-1 and enhances CD8^+^ T cell-mediated cytotoxicity, although the investigated TPC2-dependent mechanisms emphasized proliferation driven by MAPK signaling, particularly ERK1/2 [81]. Moreover, by facilitating endolysosomal degradation of GSK3β, TPC2 promotes melanoma cell proliferation, migration, and invasion by preserving β-catenin and MITF, and by directly binding to TPC2 and enhancing its channel function, Rab7a can increase this activity [82]. In endothelial cells challenged by proinflammatory factors, TPC2 activity is critical for PM trafficking of CD63 from endolysosomes via Weibel-Palade bodies, which retains P-selectin on the cell surface to facilitate leukocyte recruitment [83]. TPC1 and TPC2, when activated by NAADP, are also involved in Ca^2+^ release from cytolytic granules of cytotoxic T cells, and the Ca^2+^ nanodomains produced in this manner trigger granule exocytosis at the immunological synapse, leading to the killing of target cells [84].

TPC regulation of the endocytic pathway was first demonstrated by the impairment of endosomal trafficking of low-density lipoprotein-cholesterol and epidermal growth factor (EGF) and its receptors (EGFR) in mouse embryonic fibroblasts (MEF) and hepatocytes lacking TPC2. It was suggested that these changes most likely reflected a fusion defect between late endosomes and lysosomes, which would hinder the delivery of the substrate for degradation. It is worth noting that the loss of TPC2 did not alter endolysosomal acidification or lysosomal enzyme function in these cells [43,85,86], which contrasts with other reports claiming effects of TPC2 on lysosomal pH and degradation capacity [58,87,88]. Evidence also exists for TPC1 to regulate EGFR endosomal trafficking, but at different stages than TPC2, owing to their distributions in different endolysosomal populations [86]. Additionally, TPC inhibition disrupts β1-integrin recycling in cancer cells, resulting in its accumulation in EEA1-positive early endosomes; this function of TPCs appears to be critical for metastatic invasion of various cancers [89]. The results presented in these papers argue for a facilitating role of TPC1 and TPC2 along various steps of endocytic trafficking, including not only cargo delivery to the lysosome for degradation but also recycling of receptors back to the PM [43]. Indeed, there are multiple points along the endocytic pathway where substrates taken up by endocytosis can exit the pathway before reaching the lysosome, allowing sorted cargoes to enter the cytosol, be retrogradely transported to the trans Golgi network or ER, or be recycled back to the PM [90,91]. TPC1 and TPC2 may exert their effects at different crossroads along this complex network by facilitating a certain passage over others.

**Figure 4:**
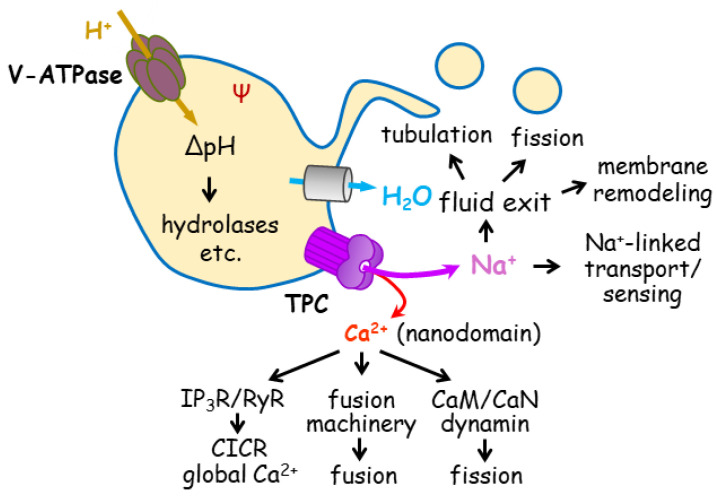
Ionic mechanism of TPC regulation of endolysosomal trafficking. By conducting Ca^2+^ and Na^+^ release, TPC impacts luminal pH and membrane potential (Ψ) of the endolysosome. Both Ca^2+^ and Na^+^ can have multiple effects on cellular functions. CaM: calmodulin, CaN, calcineurin, CICR: Ca^2+^-induced Ca^2+^ release, IP_3_R: IP_3_ receptor, RyR: ryanodine receptor.

Importantly, enveloped viruses, including filoviruses (e.g., Ebola virus) and coronaviruses (e.g., SARS-CoV-2 and MERS), exploit TPC1 and TPC2 functions within the endolysosomal system to evade digestion in the lysosome [62,92,93,94]. After entering the host cell via endocytosis, these viruses traffic through endosomes, heading eventually towards lysosomes if they do not change course. Therefore, at a certain point along this pathway, the virus must escape the endolysosomal system in order to mount a successful infection. There are at least two critical steps for viral escape: (1) cleavage of viral glycoprotein by endosomal proteases like cathepsins B and L, which relies on the low luminal pH of the endosome, and (2) fusion of the viral envelope with the endolysosomal membrane, which allows the viral genome to be released to the host cell’s cytoplasm for replication [94]. This process of viral uncoating requires assistance from cellular factors, like TPCs. However, it remains unclear how and at which step(s) TPC1 and TPC2 facilitate viral trafficking and uncoating. Clarifying the basic mechanisms of TPC regulation of endolysosomal trafficking should unveil how these channels contribute to viral infection and other (patho)physiological functions. Generally, TPCs exert three basic effects that underscore their regulation of endolysosomal trafficking (Figure 4):

Ca^2+^

Because of their role as Ca^2+^ release channels in the acidic organelles, TPCs have been proposed to produce cytosolic Ca^2+^ signals needed to trigger fusion and/or fission of endolysosomal membranes, which are essential for intracellular vesicle trafficking [86,95]. Both fusion and fission (also known as budding in some cases) are regulated by Ca^2+^ [96,97], and these two reactions are well-coordinated to regulate the delivery of sorted cargoes from one compartment to another along the endocytic and autophagic pathways. Based on their subcellular localization and studies using knockout mice, TPC1 may regulate the formation of early endosomes, i.e., budding from the PM, or fusion of the early endosomes with recycling and late endosomes or other intracellular organelles, such as Golgi apparatus [27,43]. In line with the view that TPC1 is involved in fusion, a recent study showed that knockdown of either TPC1 or synaptotagmin 7 similarly impaired antigen presentation of intracellular mycobacterial infections by MHC-related protein 1 [47]. Synaptogmins are primary Ca^2+^ sensors of the fusion machinery that work together with SNARE proteins to drive membrane fusion. It would be natural to assume that synaptotagmin 7 could serve as a sensor of the Ca^2+^ signal produced by TPC1 to trigger fusion [74]. The lysosome-localized TPC2, on the other hand, has been implicated in late endosome-lysosome fusion [85], autophagic flux [41,87,98,99,100], and exocytosis [69,71,83,100]. In addition, proteomic results suggest that TPC1 and TPC2 are physically associated with SNARE proteins [27,85,101], components of the fusion machinery.

However, there is no functional evidence that TPC1 or TPC2 directly regulates synaptotagmin 7 or SNARE complex. At least for TPC2, both the loss and increase in its expression or function have been reported to cause lysosomal enlargement [48,81,85,88], making it difficult to determine whether TPC2 primarily contributes to fusion or fission, or both, presumably under different conditions. In macrophages, the fusion between phagosome and lysosome was found to be unaffected by deletion of both TPC1 and TPC2 [79]. In Fc-receptor-mediated phagocytosis of macrophages, lysosomal exocytosis was shown to depend on TRPML1, while dynamin activation is triggered by TPC2 [40]. Controversy also exists regarding how TPC2 regulates autophagy, as both positive and negative effects of TPC2 inhibition, as well as activation, have been reported [41,87,98,99,100,102,103]. Even within the same study, TPC2 silencing was found to suppress autophagy in primary melanoma cells but enhance it in metastatic melanoma cells [99], strongly suggesting that TPC2 function may be highly context-dependent, i.e., on cell type, disease state, specific drug, treatment time, and treatment conditions.

Alternatively, TPCs could exert their primary effect on membrane fission. Because balanced fusion and fission are engaged in endolysosomal trafficking, proteins involved in fusion could indirectly exert an effect on fission and vice versa. For instance, synaptotagmin 1 is a Ca^2+^ sensor for exocytosis (fusion), but it also influences the kinetics of endocytosis and synaptic vesicle size due to exocytosis-endocytosis coupling in neuronal synapses [97]. Therefore, it is possible that many of the observed deficits on endolysosomal trafficking in TPC-deficient cells resulted from impaired fission. This view is consistent with the finding that dynamin, CaM, calcineurin, and perhaps also PKC, are activated downstream from TPCs [44,45,46], as these proteins are known to be critical for fission or endocytic budding [97]. Moreover, PIKfyve is the critical kinase that produces PI(3,5)P_2_ [68], one of the endogenous agonists of TPCs. It is well-established that PIKfyve deficiency causes endolysosomal enlargement, supporting an essential role for PI(3,5)P_2_ in vesicle fission [104]. Although other PI(3,5)P_2_ targets, such as PROPPINs/Atg18 and TRPML1, have been shown to play important roles in PI(3,5)P_2_-dependent membrane fission [54,105], it cannot be ruled out that TPC1 and/or TPC2 also participate in this process.

Experimentally, it is difficult to distinguish between fusion and fission of intracellular vesicles due to their small size (diameters ranging from 100 to 2000 nm) and the highly dynamic nature of the fusion and fission events. The conventional method using electron microscopy is excellent in resolving endolysosomal structures, but by examining only fixed samples, it cannot inform whether any connected vesicles resulted from fusion or fission. The tubular structure of early endosomes also makes it difficult to discern any areas that had undergone fusion or fission before fixation. In fact, TPCs have been demonstrated to play pivotal roles in endomembrane tubulation and tubular network formation [78,79], which, as discussed below, may share a mechanism with fission. Future studies should exploit high-speed super-resolution live-cell imaging techniques to interrogate whether TPC1 and TPC2 are primarily involved in fission or fusion.

H^+^

The effect of TPCs on pH homeostasis of endolysosomes and its impact on various hydrolases are frequently viewed as a major mechanism for their regulation of vesicle trafficking [71,72,80,87,88]. The disruption of endolysosomal pH homeostasis may also, at least in part, account for the failure of viral infection upon TPC inhibition because of the low pH environment required for the cleavage and conformational change in viral glycoproteins [94]. Studies have shown that while overexpression or hyperactivation of TPC2 leads to acidification of lysosomes or related melanosomes [48,49], the loss or inhibition of TPC2 increases lysosomal pH [80,87,88], especially under stressed conditions [58]. However, other studies found an alkalization effect of TPC2 activation or overexpression [71,72,102,103], or no change in endolysosomal pH in TPC1/TPC2 knockout cells under basal conditions [43,58,61,79,85]. Possibly, the alkalizing effect of TPC2 strongly depends on NAADP, as it was induced by TPC2-A1-N but not TPC2-A1-P, which are believed to mimic the endogenous agonists, NAADP and PI(3,5)P_2_, respectively [71]. Previously, NAADP was reported to increase pH in acidic organelles in sea urchin egg homogenates and in endolysosomal lumen of rat pancreatic acinar AR42J cells [106,107]. Because TPC2 conducts a relatively Ca^2+^-selective current when activated by NAADP and TPC2-A1-N [72], these observations are in line with the interdependence between Ca^2+^ and H^+^ in acidic stores, a phenomenon frequently exploited to verify that these stores are the origin of Ca^2+^ signals by using V-ATPase inhibitors, such as bafilomycin A1.

In studies using TPC2-A1-P to mimic PI(3,5)P_2_, no effect was observed on endolysosomal pH [71,72]. However, the gain-of-function mutant of TPC2, which displays nearly 100-fold increase in affinity to PI(3,5)P_2_ compared with the wild-type channel, caused lysosomal acidification (from pH 4.11 to 3.85) and enlargement [49]. This discrepancy may be explained by either TPC2-A1-P not completely recapitulating the function of PI(3,5)P_2_ on TPC2 or the low nanomolar affinity (~2 nM) causing stronger, more frequent, and/or longer activation of the mutant TPC2 by basal fluctuations in PI(3,5)P_2_ levels that cannot be reproduced by an acute treatment with the low-affinity drug. As multiple mechanisms are in place to prevent over-acidification of the acidic stores [108], it may take very robust and/or long-term activations of the channel, presumably by PI(3,5)P_2_, albeit the contribution of NAADP cannot be excluded at this point, to tip the balance between the forces that induce and prevent acidification. These findings suggest that the effects of TPC1 and TPC2 on endolysosomal pH are also context-dependent, particularly with respect to the Ca^2+^ vs. Na^+^ efflux triggered by NAADP vs. PI(3,5)P_2_, respectively, representing two distinct modes of TPC function.

Na^+^

Although the luminal Na^+^ concentrations of endolysosomes can vary from 5 mM to 150 mM depending on the condition and methodology used for the estimate [57,109,110], it is typically much higher than Ca^2+^; therefore, TPC1 and TPC2 conduct large Na^+^ currents, which are more pronounced than Ca^2+^, regardless of the activation trigger. The Na^+^ efflux mediated by TPC1/TPC2 has been linked to depolarization (by ~20 mV) of the endolysosomal membrane [58]. This not only contributes to endolysosomal excitability, particularly through TPC1 [111], but may also exert an impact on the activities of many lysosomal proteins, including V-ATPase and ion channels, due to driving force and voltage sensitivity. Na^+^ may act as a counterion of proton pumping; therefore, its release facilitates endolysosomal acidification, similar to Cl^−^ uptake [112]. It has also been postulated that Na^+^ dissipation through TPC2 can lower Na^+^-dependent amino acid export from the lysosome, thus allowing accumulation of amino acids at the luminal side [113]. This effect may be important for autophagy termination during prolonged starvation when degradation becomes the primary source of acquiring building blocks, and also because mTORC1 reactivation strongly depends on luminal amino acid sensing through SLC38A9, a Na^+^-coupled amino acid transporter [114,115]. Indeed, TPC2 is important for mTORC1 reactivation during starvation of skeletal muscles [87]. Therefore, Na^+^-linked transports across endolysosomal membranes, analogous to the transports across the PM, are likely regulated by TPCs through their Na^+^ conductance.

More importantly, endolysosomal trafficking is always associated with changes in the surface area-to-volume (S/V) ratio. For a sphere to split into two smaller spheres of the same size, the S/V ratio can increase by ~40%. To avoid or cope with the mechanical stress due to hydrostatic pressure buildup associated with the S/V ratio change, the vesicle can either increase its surface area or reduce its volume. Since the membrane has a limited capacity to stretch without rupture, volume reduction, which can be achieved by dumping the luminal contents, primarily water, should be preferred. As presented in the introduction, water movement across the PM follows the movement of major solutes, i.e., Na^+^ and Cl^−^. This should also be true for endomembranes. Thus, through Na^+^ efflux, TPCs can facilitate water extrusion from endolysosomes undergoing fission, tubulation, and other forms of membrane deformation that result in an increase in the S/V ratio, thereby minimizing resistance to volume reduction due to hydrostatic pressure buildup within the limiting membrane. Supporting this idea, TPC1 has been shown to play a pivotal role in a process known as phagosome resolution [116], in which newly formed phagosomes undergo fragmentation, tubulation, and budding to generate smaller vesicles to allow recovery of lipids, digestion of engulfed materials, and restoration of the capacity of phagocytes to engulf more pathogens [78,116]. In a later study, a similar role was also established for TPC2 at a later stage, referred to as phagolysosome resolution, and speculated for autolysosome resolution in the case of autophagy and lysosome reformation in general [79]. In all these examples, TPC-mediated Na^+^ efflux is thought to promote fluid loss, which alleviates membrane tension and creates membrane curvatures to enable BAR domain-containing proteins, like BIN1 and sorting nexins, to initiate and extend tubule formation [78,79]. In addition, the electrostatic change due to the luminal [Na^+^] decrease, together with increased membrane flexibility resulting from reduced tension, can potentially alter the physical property of the glycocalyx layer of lysosomes, allowing more efficient transfer of cholesterol by NPC proteins. This response is critical for the permeabilization of phagocytosed membrane, and perhaps also the inner membrane of autophagosomes, a prerequisite for the lysosomal hydrolases to gain access to their contents for degradation [79]. This mechanism may also help explain the role of TPC2 in Ebola virus infection, as the virus hijacks both NPC1 and TPCs to evade lysosomal digestion [62,79,117].

Importantly, the loss of TPC1 and TPC2 was shown to affect neither phagosome fusion with lysosome nor phagolysosomal pH, and the effect of TPCs on phagolysosome resolution was dependent on Na^+^, but not Ca^2+^ [79]. In line with the codependence of osmosis on Na^+^ and Cl^−^, the proton-activated Cl^−^ channel (PAC/ASOR/TMEM206) was reported to serve a similar function as TPC1 in phagosome resolution [118]. Together, these findings strengthen the idea that TPCs, when partnering with Cl^−^ channels, play critical roles in fluid secretion from endolysosomes to allow membrane deformation, which may underlie many of the reported pathophysiological functions of these channels. This mechanism emphasizes the Na^+^ conductance of TPCs and its role in membrane fission and tubular network formation, which are often underappreciated in early studies. For example, in platelets, the transfer of membrane proteins and luminal contents between platelet dense granules is mediated by membranous tubules, which transiently connect the granules. Although ion flux through TPC2 was found to enrich these tubules, the study mainly focused on the role of TPC2 in Ca^2+^ release from and luminal pH of the granules [119]. It would be imperative to revisit and decipher whether this tubulation effect depends on Ca^2+^, Na^+^, or pH.

Several studies have demonstrated the roles of TPC1 and TPC2 in the kinetics of endolysosomal trafficking, rather than completely abolishing it [27,43,85,86]. This can be explained by their mechanism of action on endomembrane remodeling through Na^+^ regulation of osmosis. In the absence of this regulation, membrane deformation associated with vesiculation, tubulation, and fission will be hindered by the buildup of hydrostatic pressure due to the inability to adjust the S/V ratio quickly. The slowing of endolysosomal trafficking due to TPC deficiency suggests that these channels are more efficient in inducing fluid exit than other mechanisms of adjusting the S/V ratio. For example, TMEM63A, a mechanosensitive cation channel, mediates Na^+^ efflux, leading to water extrusion from lysosomes in response to osmotic shock [120]. However, this tubulation effect is reactive to membrane tension, contrasting to that caused by TPCs, which can be proactive, as their activation does not require a prior buildup of membrane tension. Interestingly, as mentioned above, TMEM63 is a negative regulator of TPCs [69], suggesting that these channels are not simultaneously active. Recently, it was shown that transfer of lipids from the ER is involved in lysosome enlargement and vacuole formation under stressed conditions [121]. This mechanism could also increase the S/V ratio and, in turn, support membrane deformation of endolysosomes, but the kinetics may be much slower than that mediated by TPCs.

The Na^+^-centric mechanism of TPC action does not exclude the contributions of Ca^2+^ and H^+^ on similar and/or different aspects of endolysosomal trafficking discussed above. However, for functions in which both TPC1 and TPC2 have been shown to be equally critical, e.g., viral infection and phagolysosome resolution [62,79], the Na^+^-driven fluid exit pathway likely represents the major underlying mechanism. By reducing membrane tension in respective populations of endosomes and lysosomes, TPC1 and TPC2 act in tandem to allow efficient transfer of sorted cargoes along different steps of the endocytic pathway through fission and/or tubulation. The dual activation mechanism of these channels, by PI(3,5)P_2_ and NAADP that bias Na^+^ and Ca^2+^ conductances, respectively, could serve distinct roles through segregation of the channels and enzymes that produce these endogenous ligands in different areas of the same vesicle or in different vesicles. Thus, NAADP-evoked Ca^2+^ signal could elicit fusion, while PI(3,5)P_2_-driven Na^+^ efflux support fission in a spatiotemporally segregated fashion. In a recent study demonstrating a detrimental function of TPC2 in dopaminergic neurons through its Ca^2+^ signaling in a Parkinson’s disease model, biased activation of its Na^+^ conductance was found to be corrective [122]. Therefore, deciphering the distinct roles of TPC1 and TPC2, their dependence on PI(3,5)P_2_ vs. NAADP, along with the consequent effects on Na^+^ and Ca^2+^ efflux at various stages of endolysosomal trafficking, is of great importance and represents a significant future research direction.

## 6. Concluding Remarks

Recent studies have demonstrated the significance of TPC1 and TPC2 in many physiological functions and their involvement in various diseases. These endolysosomal channels are activated by NAADP and PI(3,5)P_2_ to conduct Ca^2+^ and Na^+^ efflux from the acidic organelles. In specific pathophysiological settings, the two agonists may or may not work together, and the channel might adapt distinct conformations when activated by either agonist alone or in combination. The conducting ions could have both unique and overlapping impacts on cellular functions. Therefore, when considering the contribution of a TPC to pathophysiological regulation, both the activation mode(s) and the ionic signals involved, as well as the consequences of these signals, should be carefully evaluated.

## Data Availability

No new data were created or analyzed in this study.
